# Prevalence, Incidence and Ecological Determinants of Diabetic Retinopathy in Iran: Systematic Review and Meta-analysis

**DOI:** 10.18502/jovr.v14i3.4790

**Published:** 2019-07-18

**Authors:** Golnoush Sadat Mahmoudi Nezhad, Reza Razeghinejad, Mohsen Janghorbani, Alireza Mohamadian, Mohammad Hassan Jalalpour, Somaye Bazdar, Alireza Salehi, Hossein Molavi Vardanjani

**Affiliations:** ^1^ MPH Department, Shiraz University of Medical Sciences, Shiraz, Iran; ^2^ Poostchi Ophthalmology Research Center, Department of Ophthalmology, Shiraz University of Medical Sciences, Shiraz, Iran; ^3^ Glaucoma Division, Stein Eye Institute, David Geffen School of Medicine, University of California at Los Angeles, Los Angeles, CA, USA; ^4^ Glaucoma Service, Wills Eye Hospital, Philadelphia, PA, USA; ^5^ Department of Epidemiology and Biostatistics, School of Public Health, Isfahan University of Medical Sciences, Isfahan, Iran; ^6^ Isfahan Endocrine and Metabolism Research Center, Isfahan University of Medical Sciences, Isfahan, Iran; ^7^ Student Research Committee, Shiraz University of Medical Sciences, Shiraz, Iran

**Keywords:** Access to Health Care, Diabetic Retinopathy, Epidemiology, Human Development, Iran

## Abstract

**Purpose:**

To estimate the pooled prevalence and incidence of diabetic retinopathy (DR) in Iran and to investigate their correlations with the Human Development Index (HDI), healthcare access (i.e., density of specialists and sub-specialists), and methodological issues.

**Methods:**

Electronic databases such as PubMed, Embase, Scopus, Web of Science, Google Scholar, and local databases were searched for cohort and cross-sectional studies published prior to January 2018. Prevalence and incidence rates of DR were extracted from January 2000 to December 2017 and random effects models were used to estimate pooled effect sizes. The Joanna Briggs Institute critical appraisal tool was applied for quality assessment of eligible studies.

**Results:**

A total of 55,445 participants across 33 studies were included. The pooled prevalence (95% CI) of DR in diabetic clinics (22 studies), eye clinics (4 studies), and general population (7 studies) was 31.8% (24.5 to 39.2), 57.8% (50.2 to 65.3), and 29.6% (22.6 to 36.5), respectively. It was 7.4% (3.9 to 10.8) for proliferative DR and 7.1% (4.9 to 9.4) for clinically significant macular edema. The heterogeneity of individual estimates of prevalence was highly significant. HDI (P < 0.001), density of specialists (P = 0.004), subspecialists (P < 0.001), and sampling site (P = 0.041) were associated with heterogeneity after the adjustment for type of DR, duration of diabetes, study year, and proportion of diabetics with controlled HbA1C.

**Conclusion:**

Human development and healthcare access were correlated with the prevalence of DR. Data were scarce on the prevalence of DR in less developed provinces. Participant recruitment in eye clinics might overestimate the prevalence of DR.

##  INTRODUCTION

Diabetic retinopathy (DR) is the leading cause of vision loss in adults aged 20-74 years, and remains one of the foremost causes of blindness and visual impairment worldwide.^[1,2,3,4]^ Despite significant development in the prevention and control of DR, the proportion of DR increased by 7.7% among all declining causes of blindness between 1990 and 2015.^[5]^ The prevalence of DR strongly correlates with both the duration of diabetes and the level of glycemic control.^[6]^ Therefore, timely management of DR stemming from screening programs, appropriate referral for treatment, and improving healthcare accessibility are important in preserving vision in diabetics.^[7]^

Although the treatment of DR can decrease the risk of visual loss by 60%, it imposes a heavy cost to the healthcare system.^[8]^ Despite some improvements in diagnostic assessment and treatment options,^[6]^ the lack of qualified healthcare services along with a Westernized lifestyle have caused the burden of DR to be high and on the rise in developing countries.^[7,8]^ DR is a pressing public health matter, probably due to suboptimal access to diabetes care services such as eye care professionals and eye care services, especially in low- to middle-income countries.^[8]^ Low human development might be another correlate of the increasing burden of DR. To the best of our knowledge, no study has investigated the correlation between human development and DR. The only study on this subject assessed the association between the Human Development Index (HDI) and the number of studies published on DR.^[9]^ HDI is abstracted from income, education, and life expectancy markers and ranks areas into different levels of human development.^[10]^

Despite the high prevalence of diabetes, there are few reliable national studies on the incidence, prevalence, and correlates of DR in developing countries.^[11]^ Of note, Iran is a country in transition, having a high variety of healthcare access options and human development as well as a huge variation in the prevalence and incidence of DR across its geographic regions.^[12,13,14]^ Therefore, besides assessing the prevalence and incidence of DR, their adjusted correlations with HDI and healthcare access were investigated in the current observational study.

##  METHODS

###  Protocol and Registration

The Meta-analysis of Observational Studies in Epidemiology (MOOSE) guidelines were followed.^[15]^ The study protocol was approved by the Shiraz University of Medical Sciences (ethical approval code: IR.SUMS.MED.REC.1397.256).

###  Eligibility Criteria

Observational studies (prospective or retrospective cohort and cross-sectional) were included if they provided sufficient information about the incidence and prevalence of DR and clinically significant macular edema (CSME). No restriction was applied on the year of publication or type of diabetes, and all studies published in Persian or English language were included. These two languages covered all studies published about the Iranian population and we did not find studies in other languages.

###  Search Strategy

**Figure 1:**
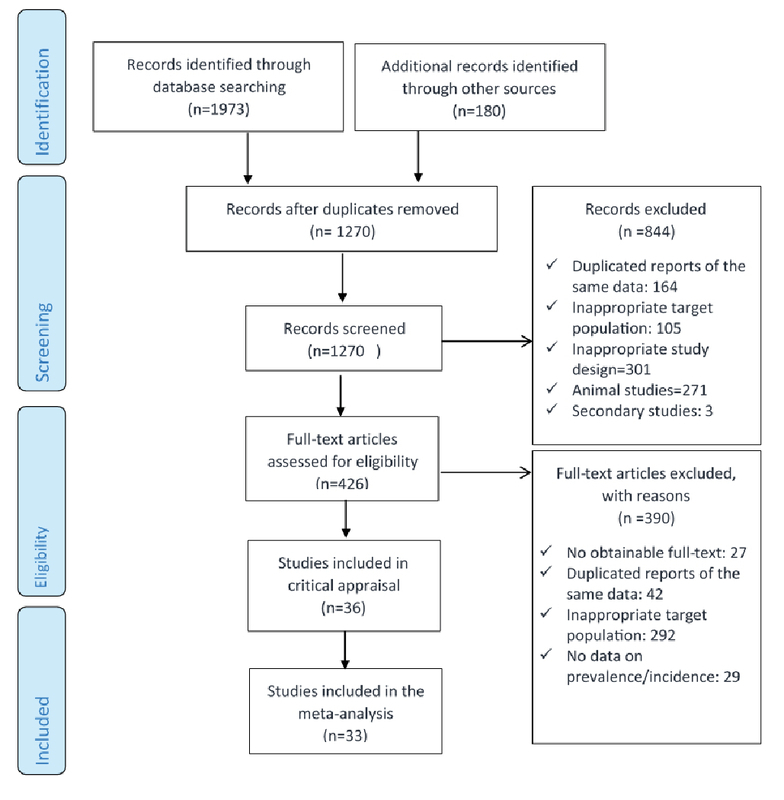
Flow diagram summarizing the systematic search and review process for identifying evidence regarding the prevalence and diabetic retinopathy. Inclusion and exclusion criteria are provided in the text.

We performed a systematic search for the prevalence and incidence of DR, summarized in Figure 1. PubMed, Scopus, Web of Science, Google Scholar, Embase, and the local databases of SID and Iran Doc were searched for articles published between January 2000 and December 2017. Our search was limited to studies related to Iran. The search terms included: “diabetes" or “diabetic" combined with "complication" or “retina" or “vision" or “visual" or “retinopathy" or “blindness" or “clinically significant macular edema" and “Iran" and “Epidemiology" or “incidence" or “prevalence" or “proportion". Review articles and their references were checked for additional studies. The gray literature evaluation was performed using international and regional congresses that were held during the study period around the world and specifically in Iran, and we selected and hand searched the abstract books that were obtainable as much as possible. We also searched university websites for thesis and reports that were related to the subject during the study period. References of all included studies were also searched for potentially eligible studies. In cases where the full text of an article was unavailable, the corresponding author was contacted. Documents were catalogued using Endnote X4.

###  Study Selection

In case of repeated publications from one study, the newest publication was included. Titles, abstracts, and the full texts of retrieved articles were reviewed independently by two experts in the field and eligible articles were selected. Studies on children, pregnant women, or non-Iranian populations were excluded as well as experimental studies, secondary studies, letters, case-reports, case-series, commentaries, and editorials. Clinical trials were also excluded. Studies with a non-representative sample or irrelevant comorbidities were also excluded. Also, in the case of disagreement regarding exclusion or inclusion of a study, a consensus was reached through discussion between the authors.

###  Data Extraction and Risk of Bias Assessment

We performed a comprehensive literature review and designed a conceptual framework. An excel (MS Office) data sheet based on clinical principles was prepared for data extraction using our framework. The following variables were included in the data extraction form: first author, publication year, study year, study location (i.e., province/district), urbanization ratio (number of participants from the urban area/number of participants from the rural area; based on the data presented in each individual study), sampling site (diabetes clinic, eye clinic, or general population), sampling design (random, multistage, convenient, unknown), study design (cross-sectional, cohort), sample size (overall and for subgroups), age range (or mean age), duration of study, gender (or female/male ratio), diagnostic methods of DR,^[16]^ proportion of diabetics (in population-based studies), type of diabetes (or type I/type II ratio), type of DR (i.e., non-proliferative diabetic retinopathy [NPDR]) and its stage (mild, moderate, or severe), proliferative diabetic retinopathy (PDR), CSME, mean HbA1C (overall, among DR patients, and among non-DR patients), number of DR patients (or estimated prevalence and its standard error), number of incident cases of DR (or cumulative incidence rate; only for cohort studies), number of person-years of follow-up (in cohort studies), duration of DM (overall, among DR patients, and among non-DR patients), mean age at the onset of DM, mean duration of DR, and proportion of patients with newly diagnosed DM. The aforementioned data were extracted (if available) and 25% of the extracted data were randomly cross-checked by another author. Also, in the case of disagreement regarding data extraction, a consensus was reached through discussion between the authors. Since several studies had not reported exact values for some variables, more than 30 disagreements on the most appropriate estimates for these missing values were discussed in the team.

The Joanna Briggs Institute (JBI)'s critical appraisal tool^[17]^ was applied for quality assessment of eligible studies. Quality assessments and critical appraisals were performed by two different authors independently. In the case of disagreement regarding the quality score, a consensus was reached through discussion between the authors (4 studies out of the included 33 studies).

###  Data on Human Development and Healthcare Access

The density of specialists and subspecialists (numbers of specialists and subspecialists in the healthcare system of each province to the total population in that province) was retrieved from a recent reliable report by Haghdoost et al^[18]^ as indices of healthcare access. Density ratios were categorized into quantiles. Haghdoost et al gathered the number of physicians based on the questionnaires filled out by medical universities all around Iran. Their study was a part of a project to define the national treatment map of Iran in 2025 (Naghsh-e Rahe Darman-e Iran). In order to control the precision of completing the questionnaire, their data was cross-checked with the Medical Registry Information System and different medical insurance companies.^[18]^

HDI indices (less developed, moderately developed, and developed) were retrieved for each province according to the study performed by Safaeipour et al^[12]^ and used as an independent variable in meta-regression modeling.

###  Data Preparation and Statistical Analysis

Point estimates of prevalence were extracted or calculated as the number of patients with DR divided by the number of patients with DM. Additionally, prevalence estimates of DR in the population, regardless of diabetic status, were estimated as the number of participants with DR divided by the number of total participants in population-based studies. A 95% confidence interval (CI) for all individual point estimates of prevalence was estimated where it was not mentioned.^[19]^ Cumulative incidence proportions (per 100 person-years) were calculated as the number of new DR cases divided by the number of at-risk person-years (i.e., number of study subjects multiplied by number of follow-up years).

To ensure the independence of point estimates in primary studies as well as to prevent repeated counting of participants in primary studies, only one of the overall or subgroup point estimates of each primary study was included in the meta-analysis and meta-regression models. The heterogeneity of individual estimates of prevalence was assessed according to the I-square statistic above 50%.^[20]^ In cases of high heterogeneity, correlates were conceptualized. Then, the significance and magnitude of their correlation were investigated using the random-effects meta-regression technique.

Subgroup analyses were performed according to the most important correlates of heterogeneity if applicable (i.e., sampling site, geographical location, HDI and healthcare access). Due to persistent heterogeneity (even after subgroup analysis), individual estimates of prevalence and incidence were pooled using the DerSimonian and Laird random-effects modeling method. Publication bias was investigated using Begg's and Egger's tests. Data analysis and calculations were performed using Stata software, version 11.2 (Stata Corporation, College Station, TX, USA). A two-sided P < 0.05 was considered as statistically significant. We also provided appropriate tables and graphs for showing our results (i.e., included studies, shortage in studies, methods applied for the diagnosis of DR by primary studies, study flowchart, and study forest plot by HDI categories). The study protocol was registered in the International Prospective Register of Systematic Reviews (PROSPERO), under the registration number CRD42018104626.

##  RESULTS

The initial search resulted in 2,153 records. Of these, 426 records were included in the full-text review process, and finally, 33 studies were included in the meta-analysis.

###  Overview of Included Studies 

Thirty cross-sectional and three cohort studies were included in the analysis, representing an overall number of 55,445 diabetic patients including 17,155 patients with DR [Table 1]. Among cross-sectional studies, seven had a population-based sampling design representing 24,623 participants including 5,657 diabetic patients and 2,049 patients with DR. Included cohort studies represented 1,174 diabetic patients (equivalent to 5,400 person-years) and 613 incident cases of DR.

**Table:** Characteristics of studies included in the meta-analysis

**Source**	**Study design**	**Sample size**	**Type of DM**	**No of DM **	**Site of sampling**	**Province**	**HDI**	**DM duration (year)**	**Hb A1c (mean)**	**P or I of DR (95% CI)**	**JS**
Janghorbani et al^[21]^ 2003	Coh	549	II	549	DM Clinic	Isfahan	0.724	5.8	10.6	8.9 (7.9,10.1)*	L
Manaviat et al^[22]^ 2004	C-s	590	II	590	DM Clinic	Yazd	0.7289	10.2	–	39.3 (43.2, 35.4)	M
Abdollahi et al^[23]^ 2006	C-s	181	I&II	181	DM Clinic	Tehran	0.7596	9.9	–	37.6 (44.7, 30.5)	M
Abdollahi et al^[24]^ 2006	C-s	152	II	152	DM Clinic	Tehran	0.7596	1.2	7.1	13.8 (19.3, 8.3)	M
Amini et al^[25]^ 2007	Coh	505	II	505	DM Clinic	Isfahan	0.724	10.2	7.5	14.4 (12.9, 15.9)*	M
Manaviat et al^[26]^ 2008	Coh	120	II	120	DM Clinic	Yazd	0.7289	11.6	–	11.4 (8.6, 14.2)*	H
Amini et al^[14]^ 2008	C-s	710	II	710	DM Clinic	Isfahan	0.724	0.5	9.5	9 (11.1, 6.9)	M
Gharaagaji et al^[27]^ 2008	C-s	591	I	591	Eye Clinic	Tehran	0.7596	9.7	–	37.9 (41.8, 34.0)	L
Golbahar et al^[28]^ 2008	C-s	254	II	254	Eye Clinic	Fars	0.6844	9	8.1	48.8 (54.9, 42.7)	M
Hatef et al^[13]^ 2008	C-s	4354	I&II	193	Population	Tehran	0.7596	–	–	17.0 (22.3, 11.7)	L
Hosseini et al^[29]^ 2009	C-s	3734	II	3734	Population	Isfahan	0.724	7	8.9	54.0 (55.6, 52.4)	L
Javadi et al^[30]^ 2009	C-s	7989	I&II	634	Population	Tehran	0.7596	8.9	6.9	37.8 (41.6, 34.0)	L
Soleymani et al^[31]^ 2012	C-s	140	II	140	Eye Clinic	Mazandran	0.7057	8.9	–	36.4 (44.4, 28.4)	M
Javanbakht et al^[32]^ 2012	C-s	3472	II	3472	Population	Iran	0.7	8.1	–	40.4 (42.0, 38.8)	L
Najafi et al^[33]^ 2013	C-s	243	II	243	DM Clinic	Tehran	0.7596	9.1	7.6	22.8 (28.1, 17.5)	L
Shaghaghi et al^[34]^ 2014	C-s	234	II	234	DM Clinic	West Azerbaijan	0.6436	12	–	33.3 (39.3, 27.3)	M
Yaghoobi et al^[35]^ 2014	C-s	180	II	180	DM Clinic	Tehran	0.7596	–	8.9	48.9 (56.2, 41.6)	M
Hosseini et al^[36]^ 2014	C-s	305	II	305	DM Clinic	Tehran	0.7596	8.2	–	35.7 (41.1, 30.3)	L
Maghbooli et al^[37]^ 2014	C-s	1228	II	1228	DM Clinic	Tehran	0.7596	11.3	7.5	26.6 (29.1, 24.1)	M
Tazhibi et al^[38]^ 2014	C-s	3535	II	3535	DM Clinic	Isfahan	0.724	7.1	–	53.4 (55.0 51.8)	M
Ghodsi et al^[39]^ 2014	C-s	978	II	978	DM Clinic	Fars	0.6844	10.3	9.3	10.4 (12.3, 8.5)	M
Dehghan et al^[40]^ 2014	C-s	2090	I&II	539	Population	Yazd	0.7289	–	8.8	29.5 (33.4, 25.6)	L
Azizi-Soleiman et al^[41]^ 2015	C-s	1782	II	1782	DM Clinic	Isfahan	0.724	5.8	9.1	61.7 (64.0, 59.4)	L
Shamshirgaran et al^[42]^ 2015	C-s	300	II	300	DM Clinic	Ardabil	0.6597	7.7	8.4	20.7 (25.3, 16.1)	L
Rasoulinejad et al^[43]^ 2015	C-s	1562	I&II	1562	Eye Clinic	Mazandran	0.7057	10.5	8.9	64.1 (66.5, 61.7)	H
Mehravar et al^[44]^ 2016	C-s	562	II	562	DM Clinic	Tehran	0.7596	15.6	7.9	28.1 (31.8, 24.4)	L
Valizadeh et al^[45]^ 2016	C-s	206	II	206	DM Clinic	Kerman	0.6891	13	–	45.1 (51.9, 38.3)	L
Samadi Aidenloo et al^[46]^ 2016	C-s	3010	I&II	181	Population	West Azerbaijan	0.6436	7.3	5.6	32.6 (39.4, 25.8)	L
Hashemi et al^[47]^ 2016	C-s	937	I&II	103	Population	Mazandran	0.7057	–	–	24.3 (32.6, 16)	L
Dehghane et al^[48]^ 2017	C-s	251	II	251	DM Clinic	Golestan	0.6736	8.7	8.4	51.4 (57.6, 45.2)	L
Shamshirgaran et al^[49]^ 2017	C-s	694	II	694	DM Clinic	Ardabil & East Azerbaijan	0.675	6.6	8.4	16.0 (18.7, 13.3)	L
Esteghamati et al^[11]^ 2017	C-s	30202	I&II	30202	DM Clinic	Iran	0.7	8	8.0	21.9 (22.4, 21.4)	L
Katibeh et al^[50]^ 2017	C-s	2501	I&II	535	Population	Gilan	0.6886	–	–	24.5 (28.1, 20.9)	L
c-s, cross sectional; coh, cohort; DM, diabetes mellitus; DR, diabetic retinopathy; H, high; I, incidence; P, prevalence; JBI, The Joanna Briggs Institute critical appraisal; JS, JBI Score; L, low; M, moderate; NO of DM, number of diabetics											
*incidence											

###  Assessment of Heterogeneity in Individual Estimates for Prevalence of Diabetic Retinopathy

In cross-sectional studies conducted in diabetes clinics, individual estimates for the prevalence of DR were significantly heterogeneous for overall diabetics (I-square, 99.5; P < 0.001; the heterogeneity was similar between genders [I-square, 99.4; P < 0.001]). Also, individual estimates for the prevalence of DR were highly heterogeneous in studies conducted in eye clinics (I-square, 98.0; P < 0.001) and population-based studies (I-square, 95.7; P < 0.001). Additionally, in three cohort studies, the estimates of individual cumulative incidence of DR were statistically heterogeneous with a highly significant I-square of 97.9 (P < 0.001).

###  Determinants of DR Prevalence (Correlates of Heterogeneity of Individual Estimates)

According to the random-effects meta-regression model, HDI (as an ordinal variable of tertile of HDI; adjusted OR: 0.12, 95% CI: 0.05 to 0.34, P < 0.001), density of specialists (as an ordinal variable including quintile of density ratio; adjusted OR: 1.13, 95% CI: 1.04 to 1.23, P = 0.004), density of subspecialists (as an ordinal variable including quintile of density ratio; adjusted OR: 0.85, 95% CI: 0.78 to 0.91, P < 0.001), type of DR (reference is PDR; adjusted OR: 1.30, 95% CI: 1.10 to 1.42, P < 0.001), duration of diabetes (adjusted OR: 1.05, 95% CI: 1.04 to 1.07, P < 0.001), site of study sampling (reference is population-based sampling; adjusted OR: 1.09, 95% CI: 1.02 to 1.17, P = 0.041), study year (adjusted OR: 0.97, 95% CI: 0.96 to 0.98, P < 0.001), and proportion of diabetics with controlled HbA1C (adjusted OR: 0.92, 95% CI: 0.87 to 0.97, P = 0.005) were significantly associated with the heterogeneity of individual estimates of DR prevalence.

Risk of bias was not associated with heterogeneity (P = 0.683). Type of diabetes was not a significant determinant of heterogeneity for individual estimates of DR prevalence (P = 0.10).

Due to the lack of enough data to achieve individual estimates of cumulative incidence, meta-regression modeling for cumulative incidence was not applicable.

###  Prevalence of DR among Diabetic Patients

**Figure 2:**
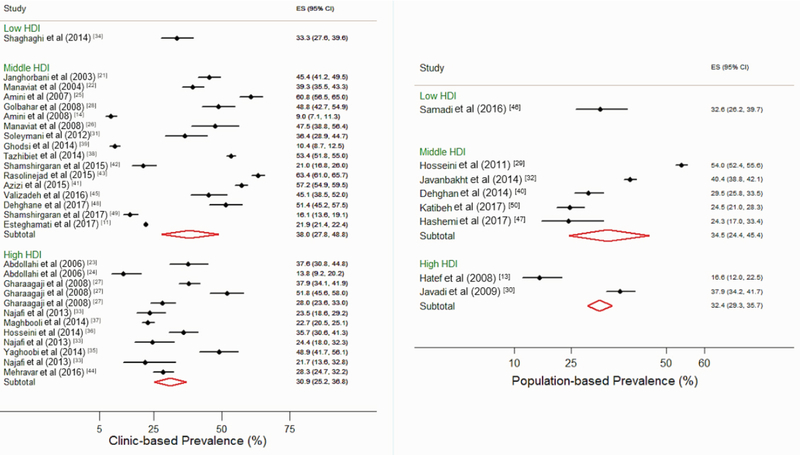
Forest plot of diabetic retinopathy prevalence by Human Development Index categories and source of data (clinic vs population-based studies). CI, confidence interval; ES, estimation of diabetic retinopathy prevalence (%); HDI, Human Development Index.

Due to high heterogeneity, pooled prevalence estimates might have some extent of bias from an epidemiological point of view. The range for overall prevalence of DR among diabetics was 9.0- 63.4%. (The pooled estimate of prevalence using random-effect model was 33.6% [95% CI: 27.9, 39.2], 40.6% [95% CI: 28.9, 52.3], and 35.7% [95% CI: 26.0, 39.4] overall in males and females, respectively [Figure 2]).

The pooled prevalence of NPDR, mild NPDR, moderate NPDR, and severe NPDR among diabetic patients were 24.8% (95% CI: 18.7, 30.9), 14.1% (95% CI: 9.1, 19.2), 8.9% (95% CI: 4.7, 13.0), and 3.3% (95% CI: 2.0, 4.6), respectively. The pooled prevalence of PDR and CSME were 7.4% (95% CI: 3.9, 10.8) and 7.1% (95% CI: 4.9, 9.4), respectively.

Due to significant heterogeneity among studies, the analysis was performed through subgroup analysis based on the most important correlates of heterogeneity.

###  Prevalence of DR among Patients Referred to Diabetic Clinics

The overall pooled prevalence rate of DR among diabetic patients was 31.8% (95% CI: 24.5, 39.2) and 39.1% (95% CI: 23.5, 54.6) among male subjects and 34.6% (95% CI: 23.2, 45.9) among female subjects.

The pooled prevalence rates of NPDR, mild NPDR, moderate NPDR, and severe NPDR among diabetic patients were 21.0% (95% CI: 12.7, 29.3), 11.1% (95% CI: 3.3, 18.9), 5.6% (95% CI: 0.7, 11.9), and 2.2% (95% CI: 1.2, 3.3), respectively. The pooled prevalence rates of PDR and CSME among diabetic patients were 2.9% (95% CI: 1.3, 4.5) and 7.4% (95% CI: 6.1, 8.6).

The pooled estimate of prevalence of DR based on studies including only type II diabetics was 33.3% (95% CI: 24.4, 42.2), whereas, it was 22.6% (95% CI: 12.8, 32.3) in those with both types of diabetes.

The pooled prevalence rates of DR in the central, northeast, northwest, southeast, and southwest geographic regions of Iran were 42.9% (95% CI: 30.4, 55.3), 51.4% (95% CI: 45.2, 57.5), 26.6% (95% CI: 18.6, 34.6), 45.1% (95% CI: 40.3, 49.9), and 10.4% (95% CI: 8.7, 12.5), respectively, and 30.8% (95% CI: 26.6, 35.0) for Tehran (the capital city of Iran).

###  Prevalence of DR among Patients Referred to Eye Clinics

The overall pooled prevalence of DR among diabetic patients was 57.8% (95% CI: 50.2, 65.3) and 63.0% (95% CI: 60.4, 65.6) among males and 61.3% (95% CI: 57.0, 65.7) among females. The pooled prevalence rates of NPDR and PDR were 29.0% (95% CI: 23.4, 34.5) and 19.4% (95 % CI: 14.6, 24.3), respectively. The pooled estimate of DR prevalence was 48.8% (95% CI: 42.7, 55.0) in studies including only type II diabetics and 64.6% (95% CI: 62.2, 67.0) in studies including both types of diabetes. The pooled prevalence rates of DR in the north and southwest geographical regions of Iran were 58.4% (95% CI: 51.3, 65.5) and 48.8% (95% CI: 44.5, 53.2), respectively, and 39.1% (95% CI: 27.2, 51.0) in Tehran.

###  Prevalence of DR among Patients in Population-based Studies

The overall pooled prevalence of DR was 29.6% (95% CI: 22.6, 36.5) and 36.8% (95% CI: 30.5, 43.2) among male subjects and 29.0% (95% CI: 24.6, 33.6) among female subjects. The pooled prevalence rates of DR in the central, north, and northwest geographical regions of Iran were 29.9% (95% CI: 26.4, 33.4), 24.4% (95% CI: 21.3, 27.5), and 32.5% (95% CI: 27.7, 37.4), respectively, and 32.8% (95% CI: 22.3, 43.3) in Tehran.

###  Prevalence of DR in the General Population

The overall pooled prevalence of DR (according to the population-based studies) was 3.6% (95% CI: 2.4, 5.0) and 3.5% (95% CI: 1.5, 6.4) among male subjects and 3.6% (95% CI: 2.1, 5.6) among female subjects.

###  Cumulative Incidence Rate of DR in Diabetics

**Figure 3:**
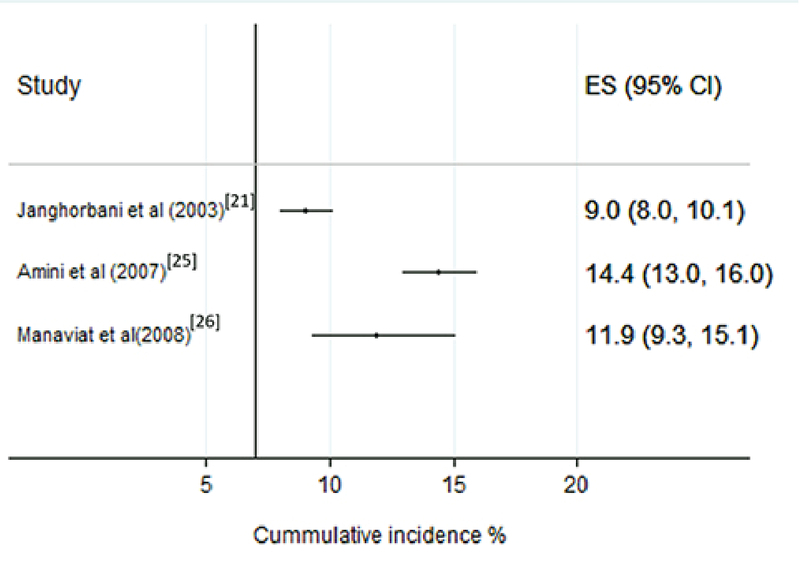
Forest plot of cumulative incidence rate of diabetic retinopathy. CI, confidence interval; ES, estimation of diabetic retinopathy incidence (%)

The overall pooled cumulative incidence rate (per 100 person-years) of DR was 11.7% (95% CI: 8.0, 15.9) and 9.6% (95% CI: 7.8, 11.8) among male subjects and 8.7% (95% CI: 7.5, 10.0) among female subjects [Figure 3]. The pooled cumulative incidence rates (per 100 person-years) for NPDR and PDR were 11.7% (95% CI: 9.1, 14.9) and 0.2% (95 % CI: 0.0, 1.1), respectively.

###  More Details on the Association of HDI and the Prevalence of DR

**Figure 4:**
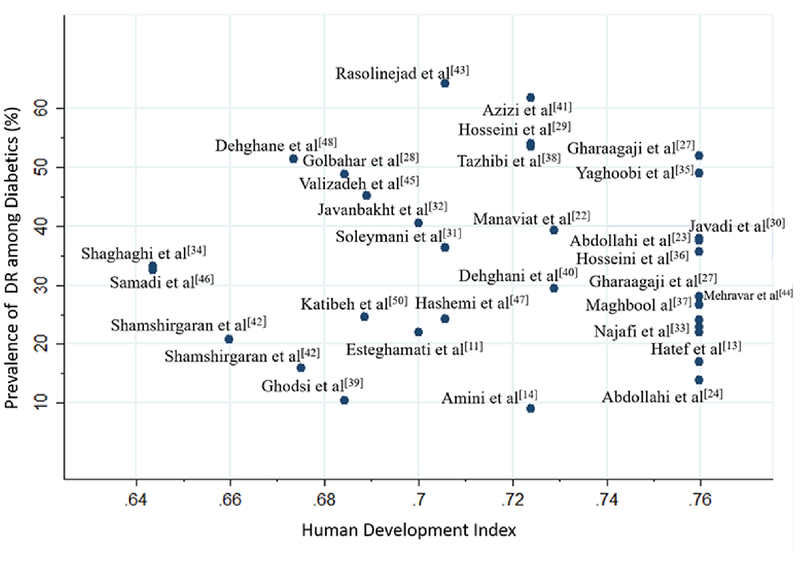
Scatter plot of the linear correlation of prevalence and the Human Development Index. DR, diabetic retinopathy

The estimation of linear correlation coefficient between HDI and the prevalence of DR among diabetics was -0.18 with a P of 0.029 (a relatively low but significant linear correlation) [Figure 4].

###  Reporting and Methodological Shortcomings

One study used the survey data analysis technique to consider multistage sampling design.^[50]^ Two studies reported age-adjusted estimates.^[30,40]^ Only 33.3% (n = 11) of the studies reported their DR results by gender [Table 2].

**Table 1:** Number (%) of studies reporting important criteria required in epidemiological studies on diabetic retinopathy


**Reportable variables**	**Studies on subjects with DM ** ***N*** ** (%)**	**Studies on subjects with DR ** ***N*** ** (%)**
Study year	26 (78.7)	26 (78.7)
Source of recruitment	33 (100)	33 (100)
Sample size	33 (100)	30 (90)
Gender	33 (100)	11 (33.3)
Age (Mean ± SD)	22 (66.6)	16 (48.4)
Dividing sample of study by age groups	1 (3.0)	1 (3.0)
Type of DM	27 (81.8)	27 (81.8)
Proportion of DM types (*n* = 11)*	2(18.2)	0
Duration of DM	14 (42.4)	11 (33.3)
Proportion of patients with controlled DM	1(3.0)	0
Type of retinopathy	14 (42.4)	14 (42.4)
Mean age at diagnosis of Diabetes	3.0 (9.0)	3.0 (9.0)
HbA1c (Mean ± SD)	20 (60.6)	15 (45)
Age-Gender adjusted prevalence of retinopathy	2 (6)	2 (6)
	
	
*Only 11 studies included both types of DM. The others reported one type of the DM in their sample size. Proportion of the DM types in these 11 studies is considered to be an important factor for both diabetics and patients with DR.		
DM, diabetes mellitus; DR, diabetic retinopathy; HbA1c, hemoglobin A1c; N, number; SD, standard deviation		

###  Assessment of DR

According to the current results, 54.54% (n = 18) of studies performed indirect ophthalmoscopy with pupillary dilatation for the evaluation of DR. Others probably used the same method; however, this matter was not clearly stated [Table 3].

**Table 2:** Findings about diagnostic methods for diabetic retinopathy


**Diagnostic method**	**Number of studies**	**Percent**
Indirect ophthalmoscopy	11	33.33
Indirect ophthalmoscopy and fluorescein angiography	3	9.09
Indirect ophthalmoscopy and fundus photography	4	12.12
Questionnaires/Records	7	21.21
Fundoscopic and angiographic findings	1	3.03
Slit-Lamp biomicroscopy of the posterior pole using contact lens	1	3.03
Eye examination and fundus photography	1	3.03
Ophthalmic examination (not mentioned exactly)	5	15.15
Total number of studies	33	100
	
	


###  Publication Bias

There was no significant publication bias (Begg's test P = 0.824; Egger's test P = 0.075) in the current study.

##  DISCUSSION

In the current study, the pooled prevalence and incidence of DR including PDR and NPDR among Iranian diabetic subjects referred to diabetes clinics and eye clinics and the pooled prevalence of DR among diabetics retrieved form population-based studies were determined based on the English language studies only. Persian articles were excluded due to poor quality score in our assessment. We found no publication bias among the included studies. The human development, access to subspecialists and specialists, and site of study sampling had a correlation with the prevalence of DR among diabetics.

Studies from other parts of the world reflect significant differences in the prevalence of DR depending on the factors such as ethnicity, demography, and access to healthcare systems.^[51,52]^ According to the current results, an increase in HDI was independently correlated with lower DR prevalence. In order to avoid the bias of ecological inference fallacy, this finding could only be interpreted from an ecological point of view. Therefore, we cannot directly relate the DR prevalence in individuals to the HDI in each province. However, HDI may potentially be a good index of quality of diabetes care in developing countries such as Iran at a national level. This finding is consistent with previous evidence from low-income regions of developed countries.^[53]^ The prevalence of DR and PDR/NPDR ratio were higher in eye clinics in comparison to the DM clinics. It might be due to the higher detection rate of DR, especially PDR in eye clinics.^[54,55,56]^ In other words, more advanced patients are usually referred to the eye clinics.

The density of subspecialists was negatively correlated with the prevalence of DR. This could be due to health literacy, greater access to healthcare services in regions where subspecialists work, leading to more effective diabetes control. This finding is in accordance with previous reports.^[51,52][57]^ Of note, many confounding factors such as social factors could be associated with the density of subspecialists and prevalence of DR. It might also be due to the interest of subspecialists to be in areas with higher socioeconomic status in which inhabitants may also have a better access to healthy food, causing more controlled DM and less prevalence of DR.

The pooled population-based prevalence of DR among diabetics was close to the maximum estimates of DR prevalence reported in developing countries such as Pakistan (29% vs 25- 29%), while it was lower than the prevalence reported in developed countries.^[58,59,60,61,62]^ Population aging and Westernized lifestyle could have been among the factors increasing the prevalence of diabetes and DR in Iran and other developing countries.^[11,58,63]^ In addition, the higher prevalence of DR among male subjects could be attributed to lifestyle habits such as cigarette smoking, which is consistent with studies conducted in India and Nepal.^[58,59]^

The pooled prevalence of DR among diabetics referred to eye clinics was significantly higher than that in diabetes clinics. Routine screening by an ophthalmologist is not a common practice in developing countries.^[64]^ Thus, this higher prevalence may be due to higher rates of referral for patients presenting with visual symptoms. There is a need for improving screening programs in primary healthcare services and communication between primary healthcare providers and ophthalmologists to ensure diabetics receive timely ophthalmic examinations.

Based on the results of the current study, the estimation of DR prevalence among diabetics in eye clinics may not be a good indicator of DR prevalence among diabetics; however, a comparison of DR prevalence in diabetic and eye clinics could help determine the sensitivity and specificity of referrals to eye clinics by clinicians and over-/underutilization of eye care in developing countries.

Although, overall and gender-specific pooled prevalence rates of DR among diabetics referred to diabetes clinics were relatively higher than those in population-based studies, these differences were not meaningful. In addition, the female/male ratio of estimated DR prevalence in DM clinics studies was almost equal to the population-based studies (88% vs 80%). Therefore, considering the difficulties in conducting robust population-based studies, it is reasonable to estimate the prevalence of DR in diabetes clinics, especially in developing counties such as Iran.

The pooled estimate of DR incidence among diabetics in Iran was less than recent reports from developed countries such as Canada, the UK, and Spain.^[61,65,66]^ Moreover, the estimated incidence was relatively higher than India, South Korea, and Denmark.^[67,68,69]^ According to the current study, the available data on the incidence of DR in Iran is inadequate; further studies are needed to determine the incidence of DR in Iran.

Among the eligible studies included in the current meta-analysis, only one study reported the prevalence of DR among Type I diabetics.^[27]^ Accordingly, the pooled estimates provided in the current study are mostly representative of the prevalence of DR among Type II diabetics. More studies on the incidence and prevalence of DR among Type I diabetics are needed.

The results of this study showed that there is a lack of research on the prevalence of DR in less developed provinces, such as Sistan and Balochistan, Bushehr, Hormozgan, Khorasan, Khozestan, Kermanshah, Kordistan, Kohkiloieye, Chaharmahal and Bakhtiyari, and Lorestan. Missing data from these provinces could have affected the results of the current study. In addition to the paucity of data in some provinces, there is a significant heterogeneity among studies, which means that making an accurate single prevalence estimate of DR is not possible. The pooled prevalence of DR was 29%. However, the prevalence rate varied from 9% to 64.1%. Thus, it might not be completely representative of the actual DR prevalence.

In addition to the aforementioned factors, the method used for detecting DR is a major factor that can influence prevalence estimates. It is crucial to know which specific diagnostic method (i.e., dilated fundus examination, direct or indirect ophthalmoscopy, digital imaging, etc.) was used in each study.^[70]^ The ophthalmic examination methods were not adequately explained in at least 12 included studies. In the course of DR, patients might develop maculopathy with no significant change in vision and may not seek medical care. Optical coherence tomography (OCT) is an important test in the evaluation and management of diabetic macular edema and can detect subclinical macular edema. Missing OCT evaluation could lead to many diabetics remaining undiagnosed in the early stage of DR.^[71]^

The current study has some limitations. The pooling of data from different sources introduced potential sources of heterogeneity that could impact accuracy. Various studies could have different inclusion criteria, sample selection, or study protocols. For example, the sample from a diabetes clinic differs from an eye clinic or population-based studies. The pooled estimate of DR prevalence by gender in the current study was sometimes not compatible with the total prevalence in the subgroups, which was due to the lack of reporting DR by gender in some studies. Studies in which the diagnosis of DM was based on patient self-report, without lab test confirmation, could have led to an overestimation of DR prevalence due to the exclusion of undiagnosed diabetes from the study sample. Although it is desirable to have the HDI and density of specialists and subspecialists at the time of each study to calculate the correlations and perform the analyses, only two up-to-date and available studies were used for estimating the value of both mentioned variables. Also, the absence of studies from the eastern, western, and southern regions of Iran could have also affected the validity and generalizability of the current findings. Although our findings on the prevalence and incidence of DR may not be generalizable to all countries, it could be useful for developing countries, especially regions with similar socioeconomic, demographic, cultural, and geographic conditions.

##  SUMMARY

Despite the scarcity of research in less developed regions, a reasonable estimate for the prevalence of DR among Iranian diabetic subjects is around 30% (29% among female and 37% among male subjects). HDI, density of specialists and sub-specialists, and sampling site were independently correlated with the prevalence of DR in Iran. The most reliable evidence on DR prevalence is likely to be retrieved from diabetes clinics in developing countries. Furthermore, providing a list of essential items for reporting the descriptive epidemiology of DR and performing studies in less developed regions could generate stronger evidence for health policy programs.

##  Financial Support and Sponsorship

Nil.

##  Conflicts of Interest

There is no conflict of interest.
